# Supplementary material for: Healthcare Quality from the Perspective of Patients in Gulf Cooperation Council Countries: A Systematic Literature Review
Source: Healthcare (Basel). 2024 Jan 25;12(3):315. doi: 10.3390/healthcare12030315 (PMC10855039; doi:10.3390/healthcare12030315)
Supplement: Supplementary file 1 [file healthcare-12-00315-s001.zip › Supplementary File S2 search strategy.pdf]

Supplementary File S2	
**Article title:**	Healthcare Quality from the Perspective of Patients in Gulf Cooperation CouncilCountries: A Systematic Literature Review
**Author names:**	Nizar Alsubahi, Milena Pavlova, Ahmed Ali Alzahrani, Ala'eddin Ahmad, and Wim Groot
**Affiliation and e-mail address of the co-author:**	Department of Health Services Research, Care and Public Health Research Institute—CAPHRI, Maastricht University Medical Center, Faculty of Health, Medicine and Life Sciences, Maastricht University, P.O. Box 616, 6200 MD Maastricht, The Netherlands; nizar.alsubahi@maastrichtuniversity.nl<br>Department of Health Service and Hospital Administration, Faculty of Economics and Administration, King Abdul Aziz University, Jeddah 21589, Saudi Arabia; nalsubahi@kau.edu.sa
**Caption:**	This file includes the search strategy and searches terms used in the database

The exact search terms used in databases are the following: ("quality of care" OR "quality of healthcare" OR "quality of health care" OR "quality of health services" OR "quality of healthcare services" OR "healthcare quality" OR "health care quality" OR "Health service quality" OR "healthcare service quality" OR "safe" OR "safety" OR "effective" OR "effectiveness" OR "timely" OR "timeliness" OR "efficient" OR "efficiency" OR "equitable" OR "equitability") AND ("person-centered" OR "person-centred" OR "patient-centered" OR "patient-centred" OR "patient-centeredness" OR "patient-centredness" OR "family-centered" OR "family-centred" OR "people-centered" OR "people-centred" OR "relationship-centered" OR "relationship-centred" OR "client-centered" OR "client-centred" OR "person-directed" OR "person directed" OR "individualized care" OR "individualised care") AND ("Saudi Arabia" OR "Kuwait" OR "United Arab Emirates" OR "Qatar" OR "Bahrain" OR "Oman" OR "GCC countries" OR "Gulf cooperation council").

Search strategy 1

(“quality of care” OR “quality of healthcare” OR “quality of health care” OR “quality of health services“ OR “quality of healthcare services“ OR “healthcare quality” OR “health care quality” OR “Health service quality“OR “healthcare service quality”) AND Saudi Arabia

(“quality of care” OR “quality of healthcare” OR “quality of health care” OR “quality of health services“ OR “quality of healthcare services“ OR “healthcare quality” OR “health care quality” OR “Health service quality“OR “healthcare service quality”) AND Kuwait

(“quality of care” OR “quality of healthcare” OR “quality of health care” OR “quality of health services“ OR “quality of healthcare services“ OR “healthcare quality” OR “health care quality” OR “Health service quality“OR “healthcare service quality”) AND United Arab Emirates

(“quality of care” OR “quality of healthcare” OR “quality of health care” OR “quality of health services“ OR “quality of healthcare services“ OR “healthcare quality” OR “health care quality” OR “Health service quality“OR “healthcare service quality”) AND Qatar

(“quality of care” OR “quality of healthcare” OR “quality of health care” OR “quality of health services“ OR “quality of healthcare services“ OR “healthcare quality” OR “health care quality” OR “Health service quality“OR “healthcare service quality”) AND Bahrain

(“quality of care” OR “quality of healthcare” OR “quality of health care” OR “quality of health services“ OR “quality of healthcare services“ OR “healthcare quality” OR “health care quality” OR “Health service quality“OR “healthcare service quality”) AND Oman

(“quality of care” OR “quality of healthcare” OR “quality of health care” OR “quality of health services“ OR “quality of healthcare services“ OR “healthcare quality” OR “health care quality” OR “Health service quality“OR “healthcare service quality”) AND GCC countries

Search strategy 2

(“person-centered” OR “person-centred” OR “patient-centered” OR “patient-centred” OR “patient-centeredness” OR “patient-centredness” OR “family-centered” OR “family-centred” OR “people-centered” OR“people-centred” OR “relationship-centered” OR “relationship-centred” OR “client-centered” OR “client-centred” OR “person-directed” OR “person directed” OR “individualized care” OR “individualised care”) AND(“Saudi Arabia” OR “Kuwait” OR “United Arab Emirates” OR “Qatar” OR “Bahrain” OR “Oman”)

(“person-centered” OR “person-centred” OR “patient-centered” OR “patient-centred” OR “patient-centeredness” OR “patient-centredness” OR “family-centered” OR “family-centred” OR “people-centered” OR“people-centred” OR “relationship-centered” OR “relationship-centred” OR “client-centered” OR “client-centred” OR “person-directed” OR “person directed” OR “individualized care” OR “individualised care”) AND(Saudi Arabia)

(“person-centered” OR “person-centred” OR “patient-centered” OR “patient-centred” OR “patient-centeredness” OR “patient-centredness” OR “family-centered” OR “family-centred” OR “people-centered” OR“people-centred” OR “relationship-centered” OR “relationship-centred” OR “client-centered” OR “client-centred” OR “person-directed” OR “person directed” OR “individualized care” OR “individualised care”) AND(Kuwait)

(“person-centered” OR “person-centred” OR “patient-centered” OR “patient-centred” OR “patient-centeredness” OR “patient-centredness” OR “family-centered” OR “family-centred” OR “people-centered” OR“people-centred” OR “relationship-centered” OR “relationship-centred” OR “client-centered” OR “client-centred” OR “person-directed” OR “person directed” OR “individualized care” OR “individualised care”) AND(United Arab Emirates)

(“person-centered” OR “person-centred” OR “patient-centered” OR “patient-centred” OR “patient-centeredness” OR “patient-centredness” OR “family-centered” OR “family-centred” OR “people-centered” OR“people-centred” OR “relationship-centered” OR “relationship-centred” OR “client-centered” OR “client-centred” OR “person-directed” OR “person directed” OR “individualized care” OR “individualised care”) AND(Qatar)

(“person-centered” OR “person-centred” OR “patient-centered” OR “patient-centred” OR “patient-centeredness” OR “patient-centredness” OR “family-centered” OR “family-centred” OR “people-centered” OR “people-centred” OR “relationship-centered” OR “relationship-centred” OR “client-centered” OR “client-centred” OR “person-directed” OR “person directed” OR “individualized care” OR “individualised care”) AND(Bahrain)

(“person-centered” OR “person-centred” OR “patient-centered” OR “patient-centred” OR “patient-centeredness” OR “patient-centredness” OR “family-centered” OR “family-centred” OR “people-centered” OR “people-centred” OR “relationship-centered” OR “relationship-centred” OR “client-centered” OR “client-centred” OR “person-directed” OR “person directed” OR “individualized care” OR “individualised care”) AND(Oman)

(“person-centered” OR “person-centred” OR “patient-centered” OR “patient-centred” OR “patient-centeredness” OR “patient-centredness” OR “family-centered” OR “family-centred” OR “people-centered” OR“people-centred” OR “relationship-centered” OR “relationship-centred” OR “client-centered” OR “client-centred” OR “person-directed” OR “person directed” OR “individualized care” OR “individualised care”) AND(Gulf cooperation council countries)

Search strategy 3

(“quality of care” OR “quality of healthcare” OR “quality of health care” OR “quality of health services“ OR “quality of healthcare services“ OR “healthcare quality” OR “health care quality” OR “Health service quality“ OR “healthcare service quality” OR “safe” OR “safety” OR “effective” OR “effectiveness” OR “timely” OR “timeliness” OR “efficient” OR “efficiency” OR “equitable” OR “equitability”) AND (“person-centered” OR “person-centred” OR “patient-centered” OR “patient-centred” OR “patient-centeredness” OR “patient-centredness” OR “family-centered” OR “family-centred” OR “people-centered” OR “people-centred” OR “relationship-centered” OR “relationship-centred” OR “client-centered” OR “client-centred” OR “person-directed” OR “person directed” OR “individualized care” OR “individualised care”) AND (“Saudi Arabia” OR “Kuwait” OR “United Arab Emirates” OR “Qatar” OR “Bahrain” OR “Oman”)

(“quality of care” OR “quality of healthcare” OR “quality of health care” OR “quality of health services“ OR “quality of healthcare services“ OR “healthcare quality” OR “health care quality” OR “Health service quality“ OR “healthcare service quality” OR “safe” OR “safety” OR “effective” OR “effectiveness” OR “timely” OR “timeliness” OR “efficient” OR “efficiency” OR “equitable” OR “equitability”) AND (“person-centered” OR “person-centred” OR “patient-centered” OR “patient-centred” OR “patient-centeredness” OR “patient-centredness” OR “family-centered” OR “family-centred” OR “people-centered” OR “people-centred” OR “relationship-centered” OR “relationship-centred” OR “client-centered” OR “client-centred” OR “person-directed” OR “person directed” OR “individualized care” OR “individualised care”) AND (“Saudi Arabia”)

(“quality of care” OR “quality of healthcare” OR “quality of health care” OR “quality of health services“ OR “quality of healthcare services“ OR “healthcare quality” OR “health care quality” OR “Health service quality“ OR “healthcare service quality” OR “safe” OR “safety” OR “effective” OR “effectiveness” OR “timely” OR “timeliness” OR “efficient” OR “efficiency” OR “equitable” OR “equitability”) AND (“person-centered” OR “person-centred” OR “patient-centered” OR “patient-centred” OR “patient-centeredness” OR “patient-centredness” OR “family-centered” OR “family-centred” OR “people-centered” OR “people-centred” OR “relationship-centered” OR “relationship-centred” OR “client-centered” OR “client-centred” OR “person-directed” OR “person directed” OR “individualized care” OR “individualised care”) AND (“Kuwait”)

(“quality of care” OR “quality of healthcare” OR “quality of health care” OR “quality of health services“ OR “quality of healthcare services“ OR “healthcare quality” OR “health care quality” OR “Health service quality“ OR “healthcare service quality” OR “safe” OR “safety” OR “effective” OR “effectiveness” OR “timely” OR “timeliness” OR “efficient” OR “efficiency” OR “equitable” OR “equitability”) AND (“person-centered” OR “person-centred” OR “patient-centered” OR “patient-centred” OR “patient-centeredness” OR “patient-centredness” OR “family-centered” OR “family-centred” OR “people-centered” OR “people-centred” OR “relationship-centered” OR “relationship-centred” OR “client-centered” OR “client-centred” OR “person-directed” OR “person directed” OR “individualized care” OR “individualised care”) AND (“United Arab Emirates”)

(“quality of care” OR “quality of healthcare” OR “quality of health care” OR “quality of health services“ OR “quality of healthcare services“ OR “healthcare quality” OR “health care quality” OR “Health service quality“ OR “healthcare service quality” OR “safe” OR “safety” OR “effective” OR “effectiveness” OR “timely” OR “timeliness” OR “efficient” OR “efficiency” OR “equitable” OR “equitability”) AND (“person-centered” OR “person-centred” OR “patient-centered” OR “patient-centred” OR “patient-centeredness” OR “patient-centredness” OR “family-centered” OR “family-centred” OR “people-centered” OR “people-centred” OR “relationship-centered” OR

“relationship-centred” OR “client-centered” OR “client-centred” OR “person-directed” OR “person directed” OR “individualized care” OR “individualised care”) AND (“Qatar”)

(“quality of care” OR “quality of healthcare” OR “quality of health care” OR “quality of health services“ OR “quality of healthcare services“ OR “healthcare quality” OR “health care quality” OR “Health service quality“ OR “healthcare service quality” OR “safe” OR “safety” OR “effective” OR “effectiveness” OR “timely” OR “timeliness” OR “efficient” OR “efficiency” OR “equitable” OR “equitability”) AND (“person-centered” OR “person-centred” OR “patient-centered” OR “patient-centred” OR “patient-centeredness” OR “patient-centredness” OR “family-centered” OR “family-centred” OR “people-centered” OR “people-centred” OR “relationship-centered” OR “relationship-centred” OR “client-centered” OR “client-centred” OR “person-directed” OR “person directed” OR “individualized care” OR “individualised care”) AND (“Bahrain”)

(“quality of care” OR “quality of healthcare” OR “quality of health care” OR “quality of health services“ OR “quality of healthcare services“ OR “healthcare quality” OR “health care quality” OR “Health service quality“ OR “healthcare service quality” OR “safe” OR “safety” OR “effective” OR “effectiveness” OR “timely” OR “timeliness” OR “efficient” OR “efficiency” OR “equitable” OR “equitability”) AND (“person-centered” OR “person-centred” OR “patient-centered” OR “patient-centred” OR “patient-centeredness” OR “patient-centredness” OR “family-centered” OR “family-centred” OR “people-centered” OR “people-centred” OR “relationship-centered” OR “relationship-centred” OR “client-centered” OR “client-centred” OR “person-directed” OR “person directed” OR “individualized care” OR “individualised care”) AND (“Oman”)
